# Application and efficacy evaluation of a novel electrolytic water-disinfecting toilet for infection prevention and control in healthcare facility restrooms

**DOI:** 10.3389/fpubh.2026.1815630

**Published:** 2026-06-10

**Authors:** Fei Fang, Jingwen Li, Wenbin He, Ying Zhang, Lanhui Tan, Weiwei Xu, Hui Zhang, Bilong Feng, Ying Wang

**Affiliations:** 1 Nursing Department of Zhongnan Hospital of Wuhan University, Wuhan, Hubei, China; 2 Department of Neurosurgery, Jingmen Central Hospital, Jingmen Central Hospital affiliated to Jingchu University of Technology, Jingmen, Hubei, China; 3 Department of Infection Control, West China Hospital, Sichuan University, Chengdu, China; 4 Wuhan Weimeng Environmental Protection Technology Co., Ltd., Wuhan, Hubei, China; 5 Hubei Engineering Center for Infectious Disease Prevention, Control and Treatment, Wuhan, Hubei, China; 6 Department of Infection Control, Wuhan University, Wuhan, Hubei, China

**Keywords:** efficacy, electrolytic disinfection, healthcare facility, hypochlorous acid, microbial contamination, restroom, smart toilet

## Abstract

**Background:**

Hospital restrooms are a weak link in infection control due to high pathogen loads, poor ventilation, and limitations of conventional disinfection. This study developed a smart toilet featuring a novel electrolytic disinfection technology to assess its clinical efficacy in decontaminating restroom surfaces and air, while exploring its potential for microbial control within hospital settings.

**Methods:**

A prospective controlled experimental study was conducted in four inpatient public restrooms at Zhongnan Hospital of Wuhan University between July and August 2025. The intervention utilized an onsite electrolysis device that electrolyzed a diluted sodium chloride solution to produce neutral electrolyzed water containing hypochlorous acid as the primary biocidal agent. Bench tests confirmed that a concentration of 40 mg/L achieved a 100% kill rate against *E. coli* within 9 min. The system was integrated into smart toilets. Surface samples (*n* = 216) and air samples (*n* = 72) were collected at 0, 5, and 30 min after use. Disinfection qualification rate, bactericidal efficacy, and natural microbial decay were compared against routine cleaning.

**Results:**

The surface qualification rate was 53.7% before and 61.1% after the intervention, a difference that was not statistically significant (*p* > 0.05). The toilet seat (5 min after use) and inner bowl (30 min after use) both reached 100% compliance, whereas the flush button demonstrated limited and inconsistent bactericidal efficacy. The air sterilization rate reached 89.7% at 30 min, which was significantly higher than the natural decay rate of 49.8% in the control group (*p* < 0.05).

**Conclusion:**

This smart toilet with novel electrolytic disinfection technology improves environmental hygiene in hospital restrooms and overcomes key limitations of manual cleaning. Nevertheless, the flush button remains a high risk site because it is not directly subjected to disinfection, thereby necessitating design optimization.

## 1 Introduction

Healthcare-associated infections (HAIs) are a major global public health threat, with an incidence of approximately 7 to 15% among hospitalized patients (1). While other transmission routes are well recognized, public restrooms have long been overlooked as sources of pathogen contamination. These areas frequently harbor a variety of pathogens associated with HAIs (2), and these microorganisms can survive on dry surfaces for weeks to months (3), thereby contributing to persistent environmental contamination within healthcare settings (4, 5).

During toilet use, infectious bioaerosols and droplets generated in hospital restrooms can be transmitted through direct or indirect contact, exposing users, cleaning staff, maintenance workers, and healthcare workers to pathogens (6, 7). Flushing urine, feces, or vomit releases bioaerosols laden with pathogens; a fraction of these rapidly settles onto surfaces within one to two meters of the toilet (8), while the rest stays airborne. Quantitative microbial risk assessment has demonstrated that a single flush can aerosolize enteric pathogens at concentrations up to 29.50 ± 10.52 CFU/m^3^, measured immediately after flushing from one meter above the floor. These infection risks exceed the acceptable thresholds established by the US EPA and WHO in hospital settings with high patient turnover (9). These bioaerosols (0.3–3 μm) can spread >1.5 m above the bowl (10). Poor ventilation and air recirculation further facilitate pathogen dissemination (11). The modes of transmission include respiratory deposition via inhalation and contact with contaminated objects, specifically touching infected surfaces followed by the face. This poses an exceptionally high risk to immunocompromised patients.

Among existing interventions, closing the toilet lid before flushing does not reduce viral contamination on toilet surfaces or adjacent floors (12). Furthermore, the application of chlorine disinfectants in clinical settings is often compromised by inappropriate concentrations, inconsistent protocols, and limited efficacy, which results in persistent bacterial contamination and biofilm formation (13). Moreover, the volatilization of chlorine agents poses risks of respiratory irritation. As a supplementary measure, ultraviolet (UV) disinfection systems fail to reach shadowed areas and pose risks to human skin and eyes, limiting their use to unoccupied spaces and thus restricting daily application (14, 15). These combined factors promote prolonged microbial survival, highlighting the urgent need for comprehensive strategies to simultaneously mitigate airborne pathogens and contaminated surfaces.

Generated by electrolyzing a sodium chloride solution to produce hypochlorous acid (HOCl) as its primary biocidal agent, neutral electrolyzed water (NEW) disrupts microbial cell membranes through its high oxidation reduction potential while causing minimal irritation to human skin and mucous membranes (16). The efficacy of NEW has been demonstrated in diverse applications, including fresh produce washing (17), food processing equipment surface disinfection (18), and oral pathogen control (19), demonstrating effective reduction of microbial contamination. However, its application in hospital restroom environments remains limited. This study developed a smart toilet integrating a novel electrolytic disinfection technology. The device combines *in situ* NEW generation with intelligent toilet functions, enabling a closed loop of disinfection with precisely controlled available chlorine. Through a dual mechanism of physical cleaning and atomized microdroplets, it extends disinfection to air and surfaces in three dimensional space. Equipped with an automatic disinfection mode operating after every flush, this toilet effectively handles the frequent use typical of hospital restrooms. An actual clinical study over 21 days was conducted to assess its efficacy in controlling microbial contamination and improving environmental hygiene. This technology offers a sustainable, financially viable, and ecologically sound solution to lower exposure burdens.

## 2 Methods

### 2.1 Development of electrolyzed water disinfection equipment

This study independently developed a novel neutral electrolyzed water generator. At its core is a titanium alloy electrolytic cell designed without a membrane, consisting of a titanium anode coated with platinum and a titanium alloy cathode. At the anode, chloride ions (Cl^−^) are oxidized to chlorine gas (Cl₂), which then hydrolyzes to form hypochlorous acid (HOCl) and other chlorine containing oxidants. At the cathode, water is reduced to hydrogen gas (H₂) and hydroxide ions (OH^−^). In this membrane free reactor, the anodic and cathodic products neutralize each other, finally producing neutral electrolyzed water (NEW) with pH 7.0–8.0. In this pH range, free chlorine coexists as hypochlorous acid (HOCl) and hypochlorite (OCl^−^), with HOCl as the main biocidal component. The electrolysis parameters were set as follows: constant current 0.842 A, voltage 8–10 V, electrolysis time 5–10 min, generating NEW with an available chlorine concentration of 25–80 mg/L.

### 2.2 Laboratory evaluation of disinfection efficacy

#### 2.2.1 Test materials

Test materials included electrolyzed water generator, NaCl (0.5 g NaCl, 1 g NaCl), ultrasonic nebulizer, agar plate, pH test paper, residual chlorine test paper, test indicator bacteria (ATCC 25922), 0.5% sodium thiosulfate neutralizer.

#### 2.2.2 Test method

Preparation of bacterial suspension: According to the “Method for Laboratory Testing of Disinfectant Bactericidal Efficacy”(GB/T 38502–2020), after activation, the second-generation slant culture medium of *Escherichia coli* was first inoculated into nutrient broth, then oscillated and mixed in a constant temperature oscillator at 37 °C, and cultivated for 16–24 h. It was centrifuged for 10 min at 8000 revolutions per minute. The obtained bacterial pellet was washed thrice with sterile physiological saline, then resuspended. A spectrophotometer was used to prepare 5 mL of bacterial suspension with approximately 10^8^ CFU/mL, which was stored at 4 °C for standby use.

Neutralizer validation: Six control groups (*n* = 3) verified 0.5% sodium thiosulfate neutralization efficacy and toxicity to *E. coli*.

Bactericidal efficacy test: *E. coli* suspensions (10^8^ CFU/mL) were exposed to two concentrations of neutral electrolyzed water: 25 mg/L (NEW1) and 40 mg/L (NEW2). Colony counts were measured after 3, 6, and 9 min using the pour plate method. Each experiment was performed in triplicate during the laboratory phase.

### 2.3 Clinical evaluation of the smart toilet

#### 2.3.1 The development of smart toilets

After laboratory tests determined the effective disinfection concentration of electrolyzed water, ward restrooms were renovated onsite using intelligent toilets equipped with the calibrated novel electrolytic disinfection technology. The device integrates multiple functions: *in situ* generation and automatic concentration control of electrolyzed water, seat cleaning, bowl inner wall atomized disinfection, and waste paper self degradation. During operation, following routine preflushing via control device 1, integrated control module 2 automatically generates electrolyzed water. The water is delivered through spraying device 4 to moisten the cleaning medium on paper output roller 3, which then moves circumferentially along the seat rail to physically strip contaminants from the seat surface. The used medium is dissolved and degraded by electrolyzed water at paper winding roller 5. Meanwhile, the system performs atomized disinfection of the bowl inner wall with the toilet lid open: sprayer 4 releases electrolyzed water as fine droplets, and integrated nozzles provide uniform coverage for comprehensive disinfection.

#### 2.3.2 Study design

A prospective experimental study was designed to preliminarily evaluate the clinical feasibility of this disinfection technology and its potential for improving hospital environmental hygiene. The study was conducted from July to August 2025 in the Radiotherapy and Chemotherapy Department of Zhongnan Hospital, Wuhan University. This department had 12 wards, each equipped with an independent public bathroom containing a ceramic sitting toilet. During screening, four bathrooms were excluded due to architectural constraints preventing device installation, and four were excluded because patient conditions were unsuitable for sampling. Finally, four eligible bathrooms were included and divided into an intervention group and a control group, with each group containing one 4 bed ward bathroom and one 3 bed ward bathroom.

For seven consecutive days before modification, baseline measurements were taken daily for each bathroom, including frequency of use, environmental bacterial colony counts, and patient turnover rates in the corresponding ward. A repeated measures design was adopted: each bathroom underwent sampling twice per week over a 3 week study period, collecting surface samples (inner bowl, seat, button) and air samples from standardized sites each time. Due to the visibility of the intervention, blinding was not possible for patients, healthcare workers, or on site researchers. However, the microbiological testing personnel and statistical analysts were blinded. Before the trial, all cleaning staff (who received standardized disinfection training), medical staff, and hospitalized patients (who received toilet use training) completed the same training duration and passed the assessment.

#### 2.3.3 Control group

In the control group, bathroom cleaning and disinfection followed conventional manual procedures: dedicated cleaning staff strictly adhered to the hospital’s standardized operating protocol, performing routine cleaning and disinfection twice daily at 7:00 a.m. and 2:00 p.m. This process included wiping surfaces with a 500 mg/L chlorine-containing disinfectant solution to maintain basic hygiene.

#### 2.3.4 Intervention group

**Figure 1:**
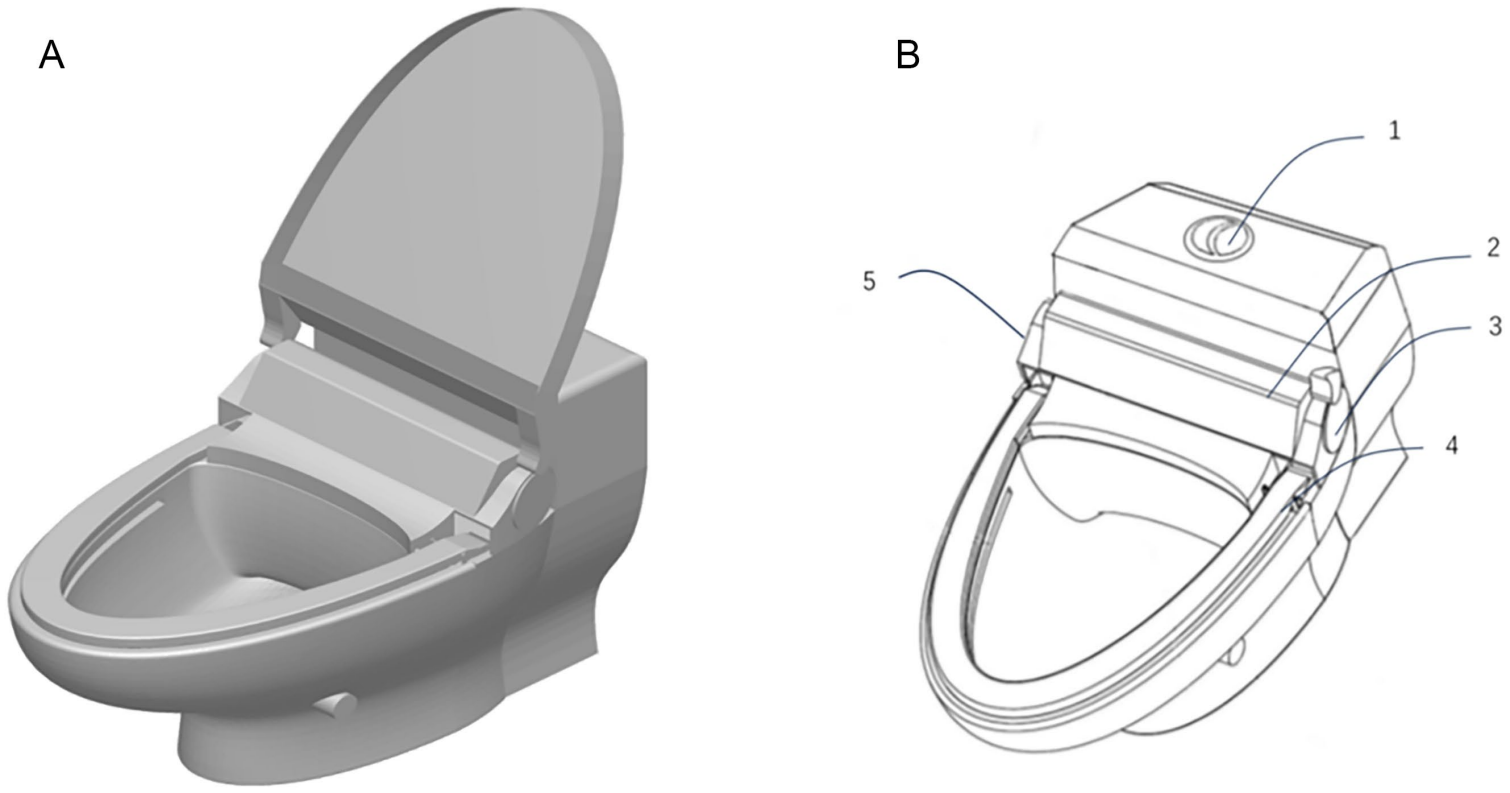
**(A)** Three dimensional and planar schematic diagrams of the smart toilet with novel electrolytic disinfection technology. Panel **(B)** is a schematic diagram of the novel electrolytic disinfection smart toilet. The numbers represent the following components: 1 flush control unit, 2 integrated control module, 3 paper output roller, 4 spray device, 5 paper winding roller.

In the intervention group, the toilet was retrofitted with the new electrolytic disinfection system before the study began. To ensure consistency with the control group, an automated disinfection cycle was established. Twice daily, at 7:00 a.m. and 2:00 p.m., the system executed a preprogrammed routine configured by the engineers. During each cycle, it automatically sprayed 200 mL of electrolyzed water directly onto the toilet seat and inner bowl surfaces. The integrated spray device operated at a flow rate of 500 mL/h, producing fine droplets with a particle size of 1–5 μm (Figure 1).

### 2.4 Specimen collection and microbiological evaluation

Prior to sampling, temperature, relative humidity, and the number of patients and caregivers in the corresponding wards were recorded for both groups. These measurements were taken to control for potential confounding factors affecting microbial load, specifically microclimate and population density. All patients were digestive cancer patients undergoing chemoradiotherapy, with a mean hospital stay of 15 days. Both groups uniformly sampled toilet seats, inner walls, and buttons as surface points. The original sampling standard (11) for object surfaces was that areas <100 cm^2^ required full coverage sampling, while those ≥100 cm^2^ required sampling of 100 cm^2^. After collection, sterile swabs were used to thoroughly elute bacterial solutions. The eluate was collected, vortex-mixed for at least 10 s, and 0.5 mL was inoculated onto agar plates for cultivation and counting. Samplers received prior training on surface and air sampling to ensure consistency and reliability.

Air sampling was performed using a six stage Andersen sampler (FA 1, Beijing Hongchangxin) to measure bioaerosol concentration after toilet flushing. The sampler flow rate was 28.3 L/min, and the sampling time was 5 min. A 90 mm nutrient agar plate (Chongqing Pangtong Medical Equipment Co., Ltd.) was placed at each stage. Before and after each sampling, the sampler was wiped with 75% alcohol cotton swabs. To control confounding from personnel movement, access was restricted, and operators entered only to retrieve the plates after sampling. The colony counts at each stage were corrected using the positive hole method, and the bioaerosol concentration was calculated in CFU/m^3^.

**Figure 2:**
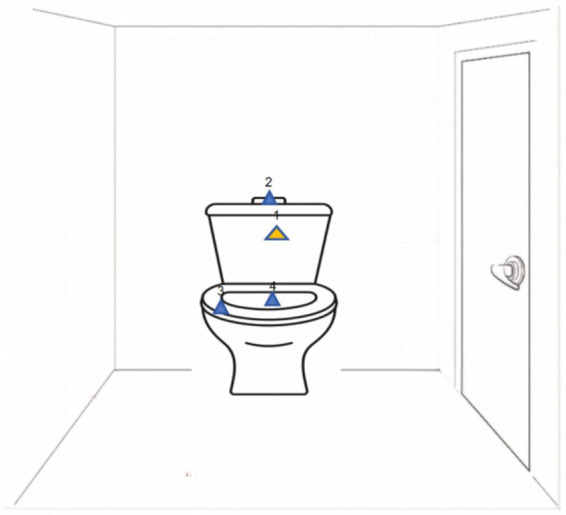
Sampling layout. The orange markers indicate air sampling points (1: 1.5 m above ground), while blue markers denote toilet surface sampling points (2: button, 3: seat, 4: inner wall).

Microbial counts on surface and air samples were determined using nutrient agar plates according to the Chinese national standard GB/T 18204.4–2013 (20). Briefly, after sample collection, 0.5 mL of the eluate was inoculated onto nutrient agar plates and evenly spread. The plates were then incubated at 37 °C in a constant temperature incubator for 24–48 h, after which colonies were observed and counted. Colony counts ranging from 30 to 300 CFU per plate were considered quantifiable; samples with fewer than 30 CFU per plate were recorded as actual counts. The bactericidal rate was calculated based on these results. Each experiment was performed in triplicate. Negative controls were included in each batch by placing one unopened petri dish directly into the incubator to verify the suitability of the culture medium for subsequent testing (Figure 2).

### 2.5 Outcomes measures

This study aimed to evaluate the advantages of the new electrolytic disinfection smart toilet compared with the cleaning method for ordinary toilets in terms of the average bacterial colony, bacterial sterilization, or natural bacterial death rates.

#### 2.5.1 Bacterial compliance rate of object surfaces

According to the hospital disinfection and sanitation standard GB 15982–2012, an object surface is deemed compliant when the average bacterial colony count is ≤10.0 CFU/cm^2^. The compliance rate is defined as the percentage of object surfaces with an average bacterial colony count ≤10.0 CFU/cm^2^ relative to the total number of object surfaces, calculated using Equation 1:


$$P=\frac{n}{N}\times 100\%$$ (1)

where *n* is the number of qualified surfaces, and *N* is the total number of surfaces examined.

Bacterial Kill (Sterilization) Rate: Measuring the effectiveness of bacterial elimination after disinfection, this is calculated using Equation 2:


$$\mathrm{KR}=\frac{A-B}{A}\times 100\%$$ (2)

where *A* is the bacterial count before disinfection, and *B* is the bacterial count after disinfection.

Natural Bacterial Death Rate: This refers to the bacterial colony count under natural conditions without disinfection, calculated using Equation 3:


$$N1=\frac{V_{0}-V_{t}}{V_{0}}\times 100\%$$ (3)

where V0 is the initial bacterial load, and Vt is the bacterial load after a specified time period.

Airborne Bioaerosol Concentration (C, CFU/m^3^): This refers to the number of viable microbial particles per unit volume of air, calculated using Equation 4:


$$C=\frac{N_{i}}{t\times 28.3}\times 1000$$ (4)

where *Ni* is the total colony count (CFU) on the sampler plates, and *t* is the sampling time (in minutes). The constant 28.3 corresponds to the sampler flow rate in L/min.

### 2.6 Data analysis

Data were organized using Excel 2023, plotted with Origin 2024, and analyzed with SPSS 26.0. Normally distributed continuous data were expressed as mean ± standard deviation, and between-group comparisons were performed using the t test. Non-normally distributed continuous data were expressed as median (M) and interquartile range (P25, P75), and between-group comparisons were performed using the Mann–Whitney U test. Repeated measures data were analyzed using the Friedman test, followed by pairwise comparisons using the Wilcoxon signed rank test with Bonferroni correction. Categorical variables were compared using the chi-square test or Fisher’s exact test, as appropriate.

## 3 Results

### 3.1 Laboratory evaluation of the bactericidal effect of neutral electrolytic water

**Figure 3:**
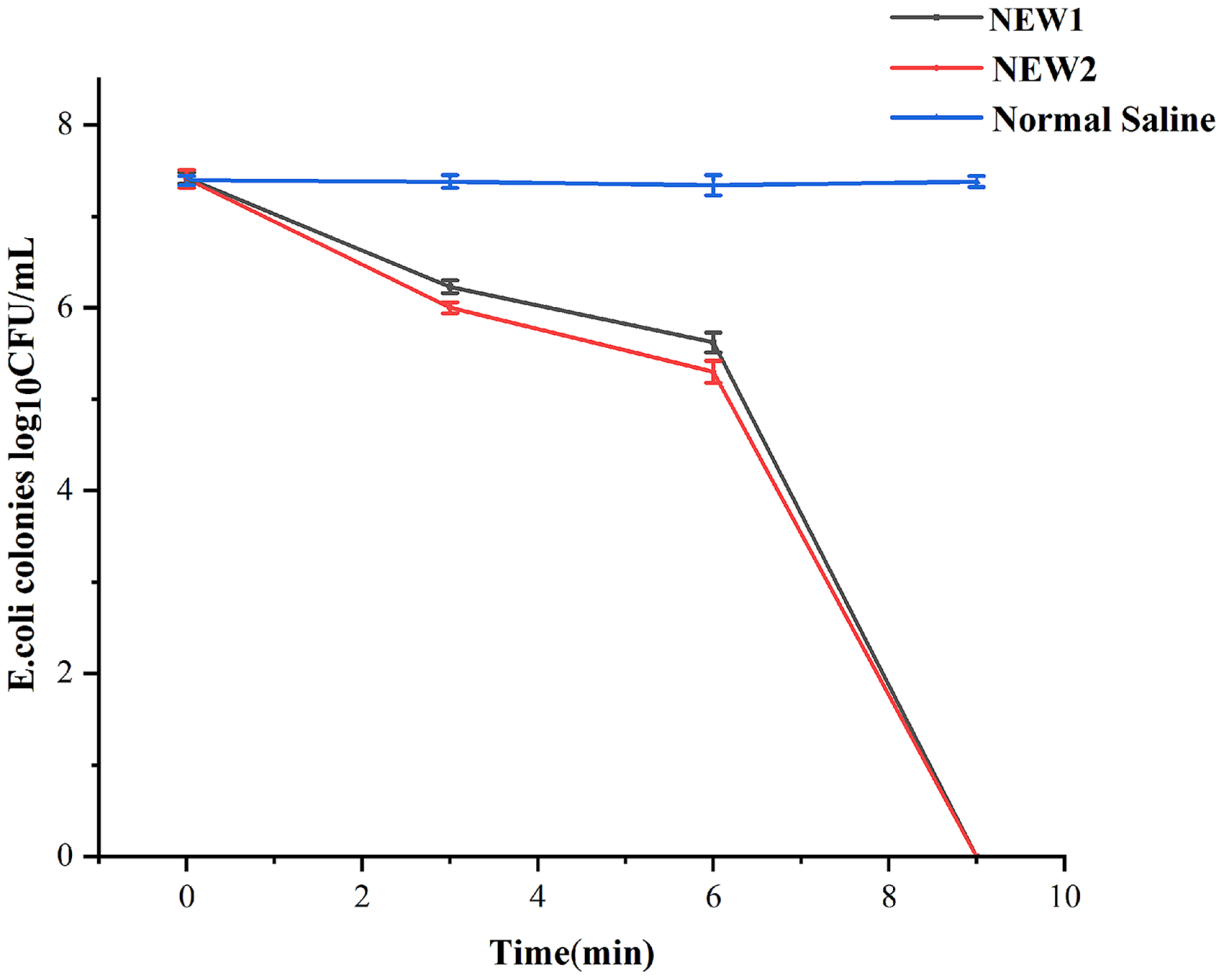
*E. coli* bactericidal effect line chart. Time-kill graph depicting the temporal dynamics of microbial load [log_10_ CFU/mL] across the experimental period (0–9 min) for NEW1, NEW2, and normal saline groups, illustrating the differential patterns of bacterial count variations over time.

The bactericidal effects of two neutral electrolyzed water solutions (NEW1 and NEW2) at different concentrations on *E. coli* are shown in Table 1. The initial mean colony counts were 7.42 ± 0.06 log_10_CFU/mL for NEW1 and 7.41 ± 0.10 log_10_ CFU/mL for NEW2. After 3 min of treatment, the viable colony count decreased to 6.23 ± 0.07 log_10_CFU/mL for NEW1 and to 6.00 ± 0.06 log_10_CFU/mL for NEW2. After 6 min, the counts further decreased to 5.62 ± 0.11 log_10_CFU/mL and 5.30 ± 0.12 log_10_CFU/mL, respectively. NEW2 demonstrated superior bactericidal effects to NEW1 at both 3 and 6 min (*p* < 0.05, *n* = 3). When the treatment time was extended to 9 min, both NEW1 and NEW2 achieved complete elimination of *E. coli* (100% kill rate), with a log reduction value exceeding 5.00. In contrast, the normal saline group exhibited no significant bactericidal effect at any time point (Figure 3).

**Table 1:** Bactericidal effect of electrolysis of water on *Escherichia coli*.

Time (min)	NEW1 (0.5 g/L)			NEW2 (1 g/L)			Normal saline		
	Viable count (log_10_CFU/mL)	Log reduction (log_10_CFU/mL)	Kill rate (%)	Viable count (log_10_CFU/mL)	Log reduction (log_10_CFU/mL)	Kill rate (%)	Viable count (log_10_CFU/mL)	Log reduction (log10CFU/mL)	Kill rate (%)
0	7.42 ± 0.06	0	0	7.41 ± 0.10	0	0	7.39 ± 0.05	0	0
3	6.23 ± 0.07	1.19	93.54%	6.0 ± 0.06*	1.41	96.11%	7.38 ± 0.07	0.01	2.28%
6	5.62 ± 0.11	1.80	98.42%	5.3 ± 0.12*	2.11	99.22%	7.34 ± 0.11	0.05	10.87%
9	ND	>5	100%	ND	>5	100%	7.38 ± 0.06	0.01	2.28%

*(*p* < 0.05) versus the NEW1 group at the same time point; ND: not detected.

### 3.2 Evaluation of sterilization effect of the new electrolytic intelligent disinfection toilet on toilet surface

Based on the laboratory data, we selected 1 g/L neutral electrolyzed water, adjusted the electrolytic device concentration to 40 mg/L, placed it inside the water tank, and installed it on the toilet. A total of 216 surface samples were collected. After using the new electrolytic disinfection smart toilet, the overall surface compliance rate was 53.7% before and 61.1% after intervention (*p* > 0.05). The electrolytic disinfection group achieved 100% compliance on the toilet seat at 5 min and on the inner bowl at 30 min. However, when compared with the chlorine disinfectant wiping group, these results showed no statistically significant differences at any time point (*p* > 0.05). The flush button showed a compliance rate of 50.0% after 30 min of intervention, indicating limited disinfection efficacy. This is likely because the device only automatically cleans and disinfects the seat and inner bowl, leaving the button without direct disinfection, and the contact time may be insufficient (Table 2).

**Table 2:** Compliance rate of bacterial colony count on bathroom surfaces after two techniques.

Toilet surface	Processing time (min)	Intervention group (*n* = 108)	Control group (*n* = 108)	*p* value
Seat	0	6/12 (50%)	7/12 (58.3%)	0.682
	5	12/12 (100%)	9/12 (75%)	0.064
	30	12/12 (100%)	11/12 (91.7%)	1.000
Inwall	0	5/12 (41.7%)	6/12 (50%)	0.682
	5	8/12 (66.7%)	7/12 (58.3%)	1.000
	30	12/12 (100%)	8/12 (66.7%)	0.093
Push-button	0	2/12 (16.7%)	1/12 (8.3%)	1.000
	5	3/12 (25%)	4/12 (33.3%)	1.000
	30	6/12 (50%)	5/12 (41.6%)	0.682

*p* value (based on χ2 test/Fisher’s exact test).

**Figure 4:**
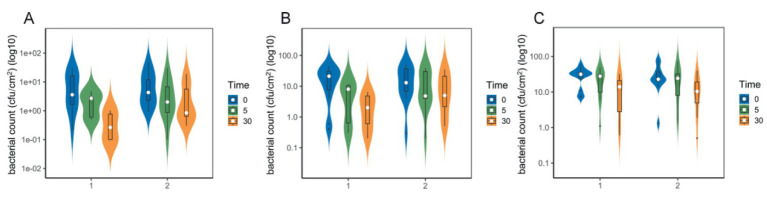
The bacterial count distribution patterns of two groups at 0, 5, and 30 minutes using a logarithmic scale. Panels: **(A)** toilet seat, **(B)** inner wall, **(C)** Push-button. Blue, green, and orange indicate 0, 5, and 30 minutes, respectively. 1 = intervention group, 2 = control group. The core trend reveals that after intervention, the intervention group showed bacterial reduction on all three surfaces, most notably on the seat and inner wall. The control group also declined but to a lesser extent. By 30 minutes, intergroup differences in median and dispersion increased for the seat and inner wall, while remaining small for the button. Overall data distribution is concentrated at low to mid values and sparse at high values, indicating predominantly low microbial loads.

The bactericidal efficacy of electrolyzed water on toilet surfaces was evaluated by comparing bacterial colony counts between the intervention group and control group at 0, 5, and 30 min (Table 3). For the toilet seat, no significant difference was observed at baseline (*p* = 0.644). After five minutes, the intervention group showed significantly lower bacterial counts than the control group (0.25 vs. 2.05 CFU/cm^2^, *p* = 0.026), with further reduction to 0 CFU/cm^2^ at 30 min (vs. 0.85 CFU/cm^2^ in controls, *p* = 0.007). For the inner wall, bacterial counts did not differ significantly at 0 or 5 min (*p* > 0.05), but were significantly lower in the intervention group after 30 min (0.75 vs. 5.0 CFU/cm^2^, *p* = 0.037). No significant between-group differences were observed for the push-button at any time point (all p > 0.05). After Bonferroni correction, bacterial counts on the toilet seat and inner wall in the intervention group were significantly lower at 5 and 30 min than at baseline (*p* < 0.05), with no significant changes between 5 and 30 min for either surface (*p* > 0.05) (Figure 4).

**Table 3:** Effects of electrolysis water treatment on total bacteria on toilet surface.

Toilet surface	Handling time	Average colony count (CFU/cm^2^)		z value	*p* value
		Intervention group (*n* = 108)	Control group (*n* = 108)		
Toilet seat	0	8.65 (1.5–21.28)	3.55 (1.78–11.58)	−0.462	0.644
	5	0.25 (0–2.35)	2.05 (0.73–9.15)	−2.221	0.026*
	30	0 (0–0.4)	0.85 (0.35–6.5)	−2.7	0.007*
Inwall	0	21.2 (5.38–31.5)	14.25 (6.25–39)	−0.058	0.954
	5	6.65 (0.4–10.75)	4.85 (4–30)	−1.156	0.248
	30	0.75 (0.05–4.35)	5 (0.7–23.5)	−2.084	0.037*
Push-button	0	31.5 (15.68–38.63)	22.9 (20.25–38.9)	−0.491	0.623
	5	28.1 (7.3–31.68)	24.85 (8–33.5)	−0.347	0.729
	30	14.9 (1.88–21.15)	10.4 (3.35–19.7)	−0.231	0.871

Data are presented as the median (interquartile range) in units of CFU/cm^2^. Between-group comparisons at each time point were performed using the Mann–Whitney U test, and the standardized test statistic (Z) is reported. *indicates a statistically significant difference (*p* < 0.05) between the intervention group and control group at the same time point.

### 3.3 Evaluation of environmental air disinfection effect of new electrolytic intelligent disinfection toilet

**Figure 5:**
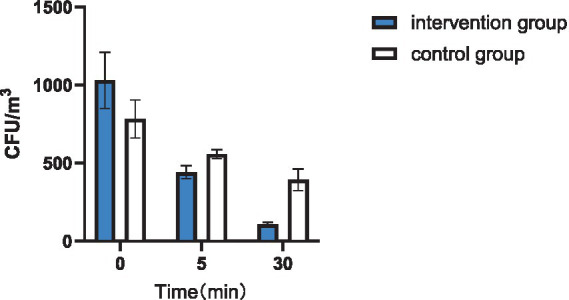
Box plot of airborne bacterial count in the bathroom. Changes in bacterial colony counts in the intervention group and control group at different time points.

During the study period, the ward temperatures ranged from 25 °C to 35 °C, and the humidity ranged from 40 to 70%. Baseline data on patient numbers and caregiver counts showed no statistically significant differences between the two groups (*p* > 0.05). The air bacterial reduction efficacy after using the novel electrolytic smart toilet is shown in Table 4. In the intervention group, the air kill rate reached 57.2% at 5 min and 89.7% at 30 min after disinfection. In the control group, the natural decay rates at the corresponding time points were 25.0 and 49.8%, respectively. Within group analysis showed that in the intervention group, the air bacterial count at 30 min was significantly lower than that at 0 and 5 min (*p* < 0.05), indicating a clear time dependent bactericidal effect. Between group comparison revealed that at 30 min, the kill rate in the intervention group was significantly higher than the natural decay rate in the control group (*p* < 0.05) (Figure 5).

**Table 4:** Effect of water electrolysis on air bacteria in two wards.

Time (min)	Intervention group (CFU/m3)	Control group (CFU/m3)	Disinfection rate (%)	Reduction rate (%)
0	1030.0 ± 623.8	782.6 ± 423.1	—	—
5	441.1 ± 142.2*	587.0 ± 95.8	57.2	25.0
30	106.2 ± 50.6*	392.8 ± 240.1	89.7	49.8

Data are presented as mean ± SD. *indicates a statistically significant difference (*p* < 0.05) between the intervention group and control group at the same time point.

### 3.4 Visualized disinfection efficacy of the new smart electrolytic toilet

**Figure 6:**
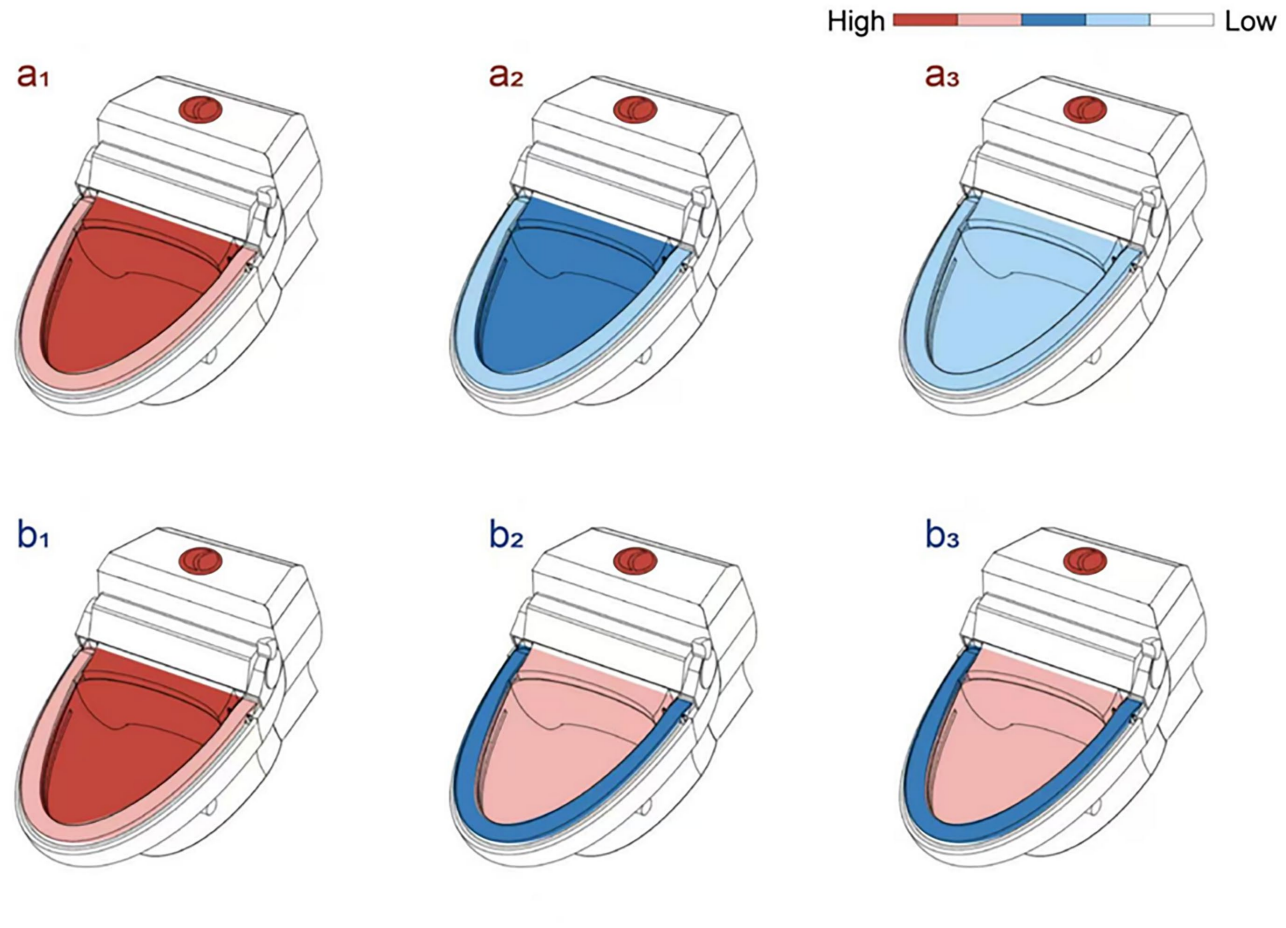
The temporal bacterial distribution gradient across toilet surfaces in the experimental and control groups. The color gradient (from dark to light) indicates bacterial abundance, with different sections of the toilet model displaying varying bacterial concentrations (Intervention group; **a1–a3**: bacterial distribution differences at 0, 5, and 30 min in the intervention group, respectively; control group; **b1–b3**: bacterial distribution differences at 0, 5, and 30 min in the control group, respectively).

Figure 6 depicts how the new electrolytic disinfection smart toilet can effectively reduce the bacterial load on the toilet surface. The seat ring and inner wall show excellent bactericidal effect, with the effect on the inner wall being enhanced with the extension of the action time, providing a feasible solution for the control of microbial pollution on the toilet surface.

## 4 Discussion

In healthcare facilities, toilets and their surrounding areas constitute high contact surfaces that exhibit widespread microbial contamination and frequently exceed permissible bacterial colony count standards (21). This study demonstrated that a toilet incorporating a novel electrolytic disinfection technology can reduce pathogen contamination levels in hospital restrooms. By operating in an automated mode, this technology achieves synergistic disinfection of the toilet seat, inner bowl, and restroom air, thereby providing a novel technical reference for environmental infection prevention and control in healthcare settings.

The present findings indicate that neutral electrolyzed water (NEW) at a higher available chlorine concentration (40 mg/L) achieves superior bacterial inactivation within a shorter contact time. Previous investigations have predominantly applied NEW in the food processing sector. Specifically, Ramirez Orejel et al. (22) reported that spraying NEW onto *E. coli* contaminated strawberries reduced the surface bacterial count to 2.12 log CFU/mL. Similarly, Jee et al. (23) showed that combined treatment with NEW and UVA substantially reduced the bioburden on stainless steel surfaces. This study represents a novel integration of a NEW generator into a smart toilet. *In situ* results revealed that following intervention, microbial loads on the toilet seat (at 5 min) and inner bowl (at 30 min) were lower than those in the control group. This outcome is attributable to the capacity of hypochlorous acid, the principal biocidal component of electrolyzed water, to penetrate microbial cells, coupled with a high oxidation reduction potential that synergistically potentiates disinfection efficacy (24). Relative to conventional chlorine based disinfectants, NEW offers advantages of safety, environmental friendliness, and lack of irritation to human skin and mucous membranes. Furthermore, the present study identified a high failure rate (53.7%) for surface cleaning and disinfection in ward public restrooms. This finding aligns with prior research; a large scale survey of 2,459 healthcare facility toilets in Bangladesh revealed that only 56% of toilets, including those in private hospitals, were judged as clean (25). Following the introduction of the electrolytic smart toilet, the difference in compliance rates between the two groups was not statistically significant. Even so, this technology offers significant potential by decreasing manual labor and providing immediate disinfection. Moreover, it delivers unique coverage benefits for challenging locations like the inner bowl and sewer pipes. Future investigations should extend the implementation to high traffic settings, including airports, schools, and parks, to further assess the economic and environmental benefits of this technology for public area cleaning and disinfection. On this basis, other green disinfection technologies, such as ozone water, could be explored for their applicability and synergistic potential within this smart toilet system to enhance stability and economic feasibility.

The surface of the toilet flush button was heavily contaminated, likely due to aerosol deposition from flushing and high frequency surface contact (26). Previous studies have confirmed that repeated exposure to a contaminated flush button increases the risk of Clostridioides difficile infection (27). However, the present study found that the novel electrolytic disinfection smart toilet did not significantly reduce the bacterial load on the flush button. This is because the device’s electrolyzed water spray only covers the seat and inner bowl, without direct disinfection of the button surface. In addition, the CT value on the button surface is insufficient; the CT value depends on the product of disinfectant concentration and contact time (28). Although the device generates electrolyzed water with an available chlorine concentration of approximately 40 ppm, the flush button is made of smooth, hydrophobic plastic, causing the spray to slide off easily and thereby reducing the actual contact time and effective CT value. These findings suggest that future designs could explore antimicrobial surface modification of the flush button or integration of button disinfection functions into the smart toilet system. Implementing these features aims to improve surface decontamination, thereby reducing the risk of infection transmission in healthcare and public environments.

With respect to air disinfection, the smart toilet incorporating novel electrolytic disinfection technology reduced the airborne bacterial load in the intervention group relative to the control group after 30 min, corresponding to a bactericidal rate of 89.7%. Prior investigations have demonstrated that a single toilet flush generates 290,000 suspended particles exceeding 0.3 μm in diameter, and individuals with their breathing zone below 1.0 m face a substantial risk of inhalation exposure even when remaining in the restroom for merely half a minute post flush (26). Consequently, interventions addressing the toilet plume should be integrated into hospital public health policies. Supporting this notion, a study conducted in South Korea reported that spraying NEW in five small animal hospitals reduced airborne bacterial concentrations by 58% after 5 min, thereby validating the air disinfection efficacy of electrolyzed water spray (29). In the present study, however, although the bacterial concentration declined at 5 min post intervention, the difference did not achieve statistical significance. A significant difference (bactericidal rate 89.7%) was observed at 30 min, attributable to the small atomized droplet size (1–5 μm) produced by the smart toilet device, which permits prolonged airborne suspension and sustained disinfection (30). Further analysis revealed that in poorly ventilated environments such as hospital restrooms, bioaerosol dispersion is relatively sluggish, and the mass concentration approximates a normal distribution (31). At 5 min, bioaerosols were predominantly concentrated in the toilet plume zone, whereas at 30 min, the spatial distribution had stabilized, indicating a cumulative disinfection effect of the device. This observation suggests that in ventilation limited settings, the evaluation time window for such disinfectants should be appropriately extended. Furthermore, relative to manual cleaning, the smart toilet equipped with novel electrolytic disinfection technology can automatically atomize and release electrolyzed water following patient use, thereby enabling immediate air disinfection and more effectively mitigating the risk of pathogen exposure.

The present study has several limitations. First, it was a single center pilot investigation with a brief observation period. No healthcare associated infections were documented during the study period, a finding that may be attributable to a temporal lag between improvements in environmental hygiene indicators and reductions in clinical infection rates. Accordingly, future research should employ multicenter methodologies spanning longer periods and utilizing large samples. Researchers should also incorporate confounding variables into multivariate analyses to ascertain the true effect of this technology on hospital infection incidence and patient outcomes. This approach will facilitate a more comprehensive and objective assessment of its clinical utility in infection prevention. Second, the present study did not quantitatively assess the spatial concentration distribution of the electrolyzed water disinfectant in the air nor the airflow pattern. These factors may influence the uniformity of disinfectant dispersion within the space, and their potential impact warrants further investigation. Third, this study did not identify bacterial species or analyze viruses. Future work could use 16S rRNA gene sequencing and mass spectrometry to identify bacteria and determine their relative abundance in restroom environments. Advanced methods such as virus isolation, culture, and real-time quantitative PCR could also be incorporated to evaluate the efficacy of electrolytic disinfection more thoroughly. Selective culture for pathogens such as Gram negative bacilli and *Staphylococcus aureus* could further demonstrate the efficacy of this disinfection technology against diverse bacteria and viruses. These combined approaches would help clarify the specific value of this technology for infection control in healthcare facility restrooms.

## 5 Conclusion

This study demonstrates that the novel electrolytic smart disinfection toilet can effectively disinfect restroom surfaces and air through an automated “use and disinfect” mode, circumventing the limitations of traditional manual disinfection and improving environmental safety. However, due to the short observation period, the long term preventive effect on hospital acquired infections and patient outcomes requires further validation with extended follow up. The flush button, which is not directly disinfected, remains a high risk point and necessitates design optimization.

## Data Availability

The original contributions presented in the study are included in the article/supplementary material, further inquiries can be directed to the corresponding authors.

## References

[1] World Health Organization. Patient safety. Geneva: World Health Organization (2019).

[2] Verhougstraete M Cooksey E Walker J Wilson AM Lewis MS Yoder A . Impact of terminal cleaning in rooms previously occupied by patients with healthcare-associated infections. PLoS One. (2024) 19:e305083. doi: 10.1371/journal.pone.0305083, 38985740 PMC11236128

[3] Porter L Sultan O Mitchell BG Jenney A Kiernan M Brewster DJ . How long do nosocomial pathogens persist on inanimate surfaces? A scoping review. J Hosp Infect. (2024) 147:25–31. doi: 10.1016/j.jhin.2024.01.023 38447803

[4] Dancer SJ. Mopping up hospital infection. J Hosp Infect. (1999) 43:85–100. doi: 10.1053/jhin.1999.0616, 10549308

[5] Dancer SJ. Controlling hospital-acquired infection: focus on the role of the environment and new technologies for decontamination. Clin Microbiol Rev. (2014) 27:665–90. doi: 10.1128/CMR.00020-14, 25278571 PMC4187643

[6] Gerba CP Wallis C Melnick JL. Microbiological hazards of household toilets: droplet production and the fate of residual organisms. Appl Microbiol. (1975) 30:229–37. doi: 10.1128/am.30.2.229-237.1975, 169732 PMC187159

[7] Leone CM Tang C Sharp J Jiang X Fraser A. Presence of human noroviruses on bathroom surfaces: a review of the literature. Int J Environ Health Res. (2016) 26:420–32. doi: 10.1080/09603123.2015.1135312, 26786956

[8] Bourouiba L. The fluid dynamics of disease transmission. Annu Rev Fluid Mech. (2021) 53:473–508. doi: 10.1146/annurev-fluid-060220-113712

[9] Abney SE Higham CA Wilson AM Ijaz MK McKinney J Reynolds KA . Transmission of viruses from restroom use: a quantitative microbial risk assessment. Food Environ Virol. (2024) 16:65–78. doi: 10.1007/s12560-023-09580-1, 38372960 PMC10963455

[10] Paddy EN Afolabi OOD Sohail M. Toilet plume bioaerosols in health care and hospitality settings: a systematic review. Am J Infect Control. (2023) 51:324–33. doi: 10.1016/j.ajic.2022.07.006, 35870658

[11] Huang Z He W Zhang L Li D Zhao Q Li Q . Assessment of sanitation facilities in primary healthcare institutions across seven provinces in China: a cross-sectional study. BMC Public Health. (2025) 25:1771. doi: 10.1186/s12889-025-22931-w, 40369471 PMC12076916

[12] Goforth MP Boone SA Clark J Valenzuela PB McKinney J Ijaz MK . Impacts of lid closure during toilet flushing and of toilet bowl cleaning on viral contamination of surfaces in United States restrooms. Am J Infect Control. (2024). 52:141–146. doi: 10.1016/j.ajic.2023.11.020 38276944

[13] Carling PC Parry MM Rupp ME Po JL Dick B Von Beheren S. Improving cleaning of the environment surrounding patients in 36 acute care hospitals. Infect Control Hosp Epidemiol. (2008) 29:1035–41. doi: 10.1086/591940, 18851687

[14] Baudart C Briot T. Ultraviolet C decontamination devices in a hospital pharmacy: an evaluation of their contribution. Pharmacy. (2025) 13:9. doi: 10.3390/pharmacy13010009, 39998007 PMC11859781

[15] Kaple CE Memic S Cadnum JL Donskey CJ. Evaluation of an automated far ultraviolet-C light technology for decontamination of surfaces and aerosolized viruses in bathrooms. Antimicrob Resist Infect Control. (2024) 13:114. doi: 10.1186/s13756-024-01473-7, 39343973 PMC11441258

[16] Chen BK Wang CK. Electrolyzed water and its pharmacological activities: a mini-review. Molecules. (2022) 27:1222. doi: 10.3390/molecules27041222, 35209015 PMC8877615

[17] Ogunniyi AD Dandie CE Brunetti G Drigo B Aleer S Hall B . Neutral electrolyzed oxidizing water is effective for pre-harvest decontamination of fresh produce. Food Microbiol. (2021) 93:103610. doi: 10.1016/j.fm.2020.103610, 32912583

[18] Jee D Ha J. Inactivation of *Escherichia coli* O157:H7, *salmonella* typhimurium, and *Listeria monocytogenes* on stainless steel by synergistic effects of tap water-based neutral electrolyzed water and lactic acid. Food Microbiol. (2023) 112:104233. doi: 10.1016/j.fm.2023.104233, 36906304

[19] Urrutia-Baca VH Paz-Michel BA Calderon-Porras AN de la Garza-Ramos MA Garcia-Garcia N Gomez-Flores R . Oral hygiene with neutral electrolyzed water and systemic therapy increases gastric *Helicobacter pylori* eradication and reduces recurrence. Clin Exp Dent Res. (2024) 10:e927. doi: 10.1002/cre2.927 38973212 PMC11228356

[20] Sun D Tang L Long K Sun W Zhang Z. Bacterial contamination in the different parts of household air conditioners: a comprehensive evaluation from Chengdu, Southwest China. Front Public Health. (2024) 12:1429626. doi: 10.3389/fpubh.2024.1429626, 39206014 PMC11350112

[21] West GF. A validation experiment: utilizing ultraviolet light to disinfect high use nursing equipment. Am J Infect Control. (2025) 53:548–51. doi: 10.1016/j.ajic.2024.12.017, 39746615

[22] Ramírez-Orejel JC Ventura-Torres P Alvarez-Cruz KA Vazquez-Hernández K Severiano-Pérez P Cano-Buendía JA. Neutral electrolyzed water as sanitizer solution in fresh foods: the strawberry as a study model. Foods. (2026) 15:800. doi: 10.3390/foods15050800, 41829072 PMC12985101

[23] Jee D Ha J. Synergistic interaction of tap water-based neutral electrolyzed water combined with UVA irradiation to enhance microbial inactivation on stainless steel. Food Res Int. (2021) 150:110773. doi: 10.1016/j.foodres.2021.110773, 34865788

[24] Hricova D Stephan R Zweifel C. Electrolyzed water and its application in the food industry. J Food Prot. (2008) 71:1934–47. doi: 10.4315/0362-028x-71.9.1934, 18810883

[25] Amin N Foster T Hossain MI Hasan MR Sarkar S Rahman A . Inadequate sanitation in healthcare facilities: a comprehensive evaluation of toilets in major hospitals in Dhaka, Bangladesh. PLoS One. (2024) 19:e0295879. doi: 10.1371/journal.pone.0295879, 38776266 PMC11111017

[26] Zhang TT Yao L Gao Z Wang F. Particle exposure risk to a lavatory user after flushing a squat toilet. Sci Rep. (2022) 12:21088. doi: 10.1038/s41598-022-25106-4, 36473899 PMC9726816

[27] Paddy EN Sohail M Afolabi OOD. Evaluating the risk of Clostridioides difficile infection from toilet flushing: a quantitative microbial risk assessment and implications for infection control. J Hosp Infect. (2025) 159:92–9. doi: 10.1016/j.jhin.2025.02.012, 40024456

[28] Wang Y Wu G Wan Q Wang J Wen G. Comparisons on the evaluation methods of chlorine resistance fungi in drinking water. Environ Res. (2025) 278:121650. doi: 10.1016/j.envres.2025.121650, 40258467

[29] Bae D Kim Y Song K Kwon J Chon J. Airborne bacterial profiling and air disinfection efficacy in small animal hospitals in South Korea. J Vet Sci. (2026) 27:e2. doi: 10.4142/jvs.25228, 41494742 PMC12891827

[30] Oikawa T Akinaga K Yanagida M Yamazaki W Sato T Mukoda Y . Effective and safe: long-term aerosol disinfection of slightly acidic electrolyzed water causes no harm in rats. PLoS One. (2026) 21:e0341050. doi: 10.1371/journal.pone.0341050, 41615984 PMC12857984

[31] Wang J Wu Z Wang H Zhong M Mao Y Li Y . Ventilation reconstruction in bathrooms for restraining hazardous plume: mitigate COVID-19 and beyond. J Hazard Mater. (2022) 439:129697. doi: 10.1016/j.jhazmat.2022.129697, 36104926 PMC9335364

